# Philadelphia County Medical Society

**Published:** 1893-01

**Authors:** 


### PHILADELPHIA COUNTY MEDICAL SOCIETY.

*Stated Meeting, November 23, 1892.*

Dr. Henry R. Wharton read a paper, entitled :

### THE TREATMENT OF ISCHIO-RECTAL ABSCESS AND FISTULA-IN-ANO.

I have been requested to say a few words this evening on the treatment of ischio-rectal abscess and fistula-in-ano to open the discussion upon these subjects by the members of this society.

*Treatment of Ischio-Rectal Abscess.*—In this form of abscess the purulent matter occupies the loose cellular tissue of the ischio-rectal fossa in close relation to the rectum, and from the anatomical peculiarities of the tissue in which it is situated it is apt to burrow widely, and, if left to itself, to open into the rectum or through the skin into the region of the anus, and result in the production of one or other forms of fistula-in-ano,—either the complete form or the external or internal incomplete form of this affection.

To obviate this unfortunate result, the prompt treatment of ischio-rectal abscess is urgently demanded, and I am decidedly of the opinion that attempts at abortive treatment of this form of abscess are worse than useless, that by such treatment valuable time is lost, and the surgeon has, finally, to resort to surgical treatment after extensive burrowing of pus has occurred, with possibly perforation of the wall of the rectum.

It is, and has been for a long time, a surgical axiom that an ischio-rectal abscess should be opened promptly, and, if so treated, the probability of a fistula-in-ano resulting is much diminished. I formerly was satisfied to open these abscesses by a small incision, evacuate the pus, and in many cases a prompt recovery took place without the formation of a fistula, but in others a fistula resulted ; whether the rectal communication was present at the time of opening, or resulted from the imperfect drainage secured by a small incision, I am unable to say, but I am sure that the results I have obtained in these cases during the last few years since I have adopted Mr. Allingham's method of dealing with these abscesses have been much more satisfactory. By this method of treatment, even in cases where I have been able to demonstrate a rectal communication at the time of the operation, I have secured healing without the formation of a permanent fistula. Therefore, in any case of inflammation of the tissues of the ischio-rectal fossa, whether the evidence of abscess be clearly demonstrated or not, I

follow the method which is recommended by Mr. Allingham, which consists in etherizing the patient and placing him in the lithotomy position, after having located the position of the indurated tissue or abscess ; and a rectal examination by means of the finger will often assist in locating the position of the abscess. A free incision, several inches in length, is made through the tissue, outside of and parallel with the fibers of the external sphincter muscle, and the incision is gradually deepened until the pus cavity is reached. It is then slit up to the length of the skin incision, and the cavity is explored with the finger, breaking down any loculi which tend to divide up the abscess cavity, and so make one cavity of the abscess. The cavity of the abscess is next washed out with a 1 : 2000 bichloride of mercury, or 1 : 60 carbolic acid solution, and is then packed with strips of iodoform gauze, and a pad of the same gauze is placed over the wound, and over this a pad of bichloride of mercury cotton is laid, and the dressing is secured in position by a T-bandage. An opium suppository is introduced into the rectum, and the bowels are kept quiet for three or four days.

The dressing is not removed for two or three days, and at this time the packing is usually loose and can be removed without difficulty, and after its removal the cavity is injected with peroxide of hydrogen, and it is then irrigated with a 1 : 2000 bichloride of mercury solution, and next the cavity is lightly packed with strips of iodoform gauze, and the wound is covered with a pad of iodoform gauze and bichloride cotton. The same steps are observed at subsequent dressings, which are made at intervals of two or three days, and the cavity usually heals rapidly by granulation and contraction, and in a few weeks it is usually completely healed.

Mr. Allingham recommends that the cavity be packed with lint saturated with carbolized oil, and I have employed this material, but now prefer to use the iodoform gauze, as I stated above.

I will report briefly a case in which this treatment was adopted. In January of this year I saw, with Dr. Musser, a lady, forty years of age, who had suffered for a few days with inflammation of the tissues of the ischio-rectal fossa. On examination of the case I found the left buttock for a distance of six or eight inches from the very verge of the anus, indurated, hot, and painful ; no soft spot of pointing could be detected. An examination of the rectum showed bulging of the walls of the rectum in the left side, and upon withdrawing the finger a small amount of pus escaped from the anus. The patient also stated that some matter had been discharged from the rectum during the day. The patient was etherized and a curved incision four inches in length was made just outside of the line of the sphincter muscle. This was gradually deepened until the cavity of the abscess was opened and a free discharge of pus, many ounces, escaped. On introducing my finger, I found that the cavity extended laterally for some distance and passed upwards between the wall of the rectum and the sacrum. In fact, with my two fingers introduced to their full length in the wound, I could not reach the upper portion of the abscess cavity. A careful examination failed to reveal the position of the opening into the rectum. The abscess cavity was thoroughly irrigated with a 1 : 2000 bichloride of mercury solution, and was then packed with strips of iodoform gauze, and a pad of gauze and bichloride cotton was placed over the external wound and held in place by a T-bandage. The patient did well after the operation, and the cavity was dressed in the same manner every second or third day for the first two weeks, and at less frequent intervals after this time for six weeks, at which time healing was complete.

There is no question in mind that there existed a communication between the abscess cavity and the rectum before the operation, as was shown by the discharge of pus, and by the discharge from the wound about a week after the operation of a piece of bone a little larger than a grain of corn. This bone Dr. Harrison Allen examined for me, and pronounced it to be a portion of a transverse process of a sheep's vertebra. It had been swallowed with the food, and had ulcerated through the wall of the rectum, and had set up inflammatory action in the peri-rectal cellular tissue, terminating in this extensive abscess.

The points in the treatment of ischio-rectal abscess I would especially call attention to are: Early and free incision; thorough breaking down of any secondary abscess cavities into one cavity; irrigation of the cavity with peroxide of hydrogen and a 1 : 2000 bichloride of mercury solution, or 1 : 60 carbolic acid solution; packing with iodoform gauze and subsequent dressings made in the same manner, care being taken not to pack the cavity too firmly. Following this form of treatment, the results of this variety of abscess in my hands have been most satisfactory.

*Treatment of Fistula-in-ano.*—Ball classifies fistula-in-ano as complete rectal fistula in which there is a sinus leading from the rectum to some point in the skin in the region of the anus; and the incomplete fistula he describes as internal rectal sinus, a sinus passing from the rectum into the peri-rectal cellular tissue ; external rectal sinus, one having an opening on the skin passing into the cellular tissue around the rectum, but not perforating the wall of the gut.

As regards the treatment of fistula-in-ano, the fact should not be lost sight of that it is possible to have a fistula-in-ano heal under simple treatment, without operative interference. This is more apt to occur in recently formed fistulæ, but, as the result of palliative treatment is always uncertain in these cases, and a long course of local applications is required, this method of treatment is not generally adopted. Allingham says that he has had twenty-one successful cases under this method of treatment, and a number of cases in which he was unable to effect a cure after prolonged treatment. When this form of treatment is adopted, it consists in trying to obliterate the fistulous tract by rest, free drainage, and the local use of stimulating applications, such as carbolic acid, nitrate of silver, and sulphate of copper. Rest to the part is best secured by the wearing of a firm anal pad secured by a T-bandage.

At the present time, the most widely adopted and successful treatment of complete fistula-in-ano is by incision. The patient is etherized and placed on his side, or in the lithotomy position, a probe-pointed flexible director is then passed through the external opening of the fistula and conducted into the rectum ; the finger is then passed through the anus until it comes in contact with the end of the director, which is bent and brought out of the anus. The tissues on the director are then divided with a scalpel or by means of scissors, care being taken to see that the division of the fibers of the sphincter muscle is made at a right angle to the course of the muscular fibers ; oblique divisions of the muscle do not heal well, and are apt to be followed by a loss of power in the muscle. The main track of the fistula being slit up, it is next explored for the presence of branching sinuses, and if these are found they are slit on a director. In indurated sinuses it is often well to make an incision through the base of the sinus, which seems, in many cases, to facilitate the healing. If the cutaneous edge of the fistula or sinuses tend to overlap each other near the anus, they should be trimmed off with scissors. The surface of the exposed fistula or sinuses is next freshened with a curette, and, after being washed out with a 1 : 2000 bichloride of mercury solution, the cavities are packed with strips of iodoform gauze or lint saturated with carbolized oil. A compress of gauze is next applied over the wound, and over this is placed a pad of bichloride cotton, and the dressing is held in place by means of a T-bandage. The patient is given an opium suppository, and the bowels are kept quiet for three or four days.

The after-treatment of fistula-in-ano is most important, and many unfavorable results are due to carelessness in this particular. On removal of the primary dressing, at the end of two or three days, the sinus should be washed out with peroxide of hydrogen and a 1 : 2000 bichloride of mercury solution, and a strip of iodoform gauze should be lightly passed to the bottom of the wound and allowed to rest between its edges. The mistake is often made in packing these wounds forcibly, which interferes with healing. A piece of gauze and a pad of cotton is next applied over the wound, and is held in place by a T-bandage. The patient should be kept on his back two or three weeks, and the wound should be dressed in the manner described, daily, or on alternate days, and at the end of three or four weeks healing is usually completed.

In cases of fistula-in-ano of the horseshoe variety, one division only of the external sphincter muscle should be made, and the branching sinuses should be laid open by curved incisions passing parallel with and outside of the line of the muscle. Sinuses extending to the perineum or buttock should be freely laid open.

The treatment of incomplete fistula of the external variety, or of external rectal sinus, consists in passing a director into the sinus down to the rectum, and if, on passing the finger into the rectum, it is found that the director is separated only from the finger by the mucous membrane, and its position is low down in the rectum, it is better to push the director into the bowel and bring it out at the anus and divide the tissues as in complete fistula, and treat the resulting wound as described after the operation for complete fistula. If, on the other hand, the rectum is merely exposed at the bottom of the sinus, it is well to lay the sinus freely open to this point, curette its surface and pack it lightly with iodoform gauze. Subsequent dressings should be carefully made and the sinus will usually heal, though the course of treatment usually extends over a longer period of time than in cases where the sphincter muscle has been divided.

In internal incomplete fistula or internal rectal sinus, when the rectal perforation is low down, a bent director should be passed into the anus, and its point should be passed through the rectal opening and made to project on the skin near the anus. This is cut down upon and exposed and the director is passed through it, making the fistula a complete one, and the tissues on the director are divided. The subsequent steps of the operation and dressing are similar to those mentioned in the cases previously described.

When the rectal opening is high up and it is considered inadvisable to divide the sphincter muscles or the bowel to its full extent, a director should be passed through the internal opening and the surgeon should cut down on its point from an incision through the skin a little outside of the sphincter muscle. When it has been exposed, the sinus or cavity should be curetted and irrigated and dressed with iodoform gauze, and, by careful dressing, the wound may be made to heal from the bottom, the rectal communication being shut off by granulation and subsequent contraction.

Among various methods of treating fistula-in-ano should be mentioned the elastic ligature and the treatment by excision.

The elastic ligature is principally used in those cases in which the fistula opens into the rectum at a high point, where division by the knife would be accompanied by free hemorrhage. When employed, a cord of india-rubber one-sixteenth of an inch in diameter is threaded to an eyed-probe, which is passed through the cutaneous opening into the rectum and brought out at the anus ; before tying the ligature, the skin and mucous membrane to the edge of the anus should be divided so that the ligation can bury itself when tied, thereby saving the patient pain, and at the same time facilitating the more rapid division of the tissues by the ligature. After the ligature has cut its way through the tissues, it is often found necessary to dress the wound in the same manner as in cases where incision has been practised, to secure satisfactory healing.

The treatment of fistula-in-ano by excision has been recommended by some surgeons. The fistulous track being dissected out, the parts are brought together by deep sutures, and if primary union is obtained, there is a great saving in the time of treatment. The form of fistula in which this method of treatment is best suited, is the complete fistulæ, which are not very deep and have a straight course ; branching fistulæ, and ones very deeply situated, I do not think are favorable cases for this procedure. If in using this method of treatment it is found that primary union has not occurred, as shown by the escape of a little pus from the line of the incision, the sutures should be removed and the edges of the wound should be separated, and it should be lightly packed and treated—in fact, as a case in which primary incision had been practised.

As fistula-in-ano often occurs in patients suffering from phthisis, the question of the advisability of operating upon these cases often must be considered. The rule in these cases is to operate, unless the patient's disease is in a very advanced state, when no repair could be likely to take place. In the majority of phthisical cases the result of the operation is satisfactory.

The only serious complication following the operation for fistula-in-ano is incontinence of feces, and this is, fortunately, a rare complication. It may be guarded against by care in dividing the sphincter muscle only at one point, and by seeing that the division of the muscle is not an oblique one. When incontinence exists, it may be relieved in many cases by excision of the cicatrix in the sphincter muscle, and by suturing the freshened ends of the muscle together by catgut sutures, or by applying a point of Paquelin's cautery to the cicatrix or to several points of the mucous membrane and skin of the anal margin.

### DISCUSSION.

Dr. Thomas S. K. Morton: I am glad that Dr. Wharton has insisted upon early operation in ischio-rectal abscess. This affection involves loose cellular tissue, richly supplied with lymphatics, and may give rise to profound septicemia, if not pyemia, if not relieved early. I have recently heard of a case of small ischio-rectal abscess which gave rise to pyemia and death. The incision should be made as soon as the brawny induration is evident, feeling certain that if there is any inflammation in this region, where the tissues are so poorly supplied with blood-vessels and other means of destroying septic material, suppuration will follow.

In washing out these abscesses with a strong antiseptic solution, it is important to see that all that goes in comes out, and that none enters the bowel, for a few drachms of a strong mercurial solution entering the bowel would give rise to poisoning.

The packing should be applied loosely. Here, as well as elsewhere, much damage can be done by too tight packing. There is something about iodoform gauze that makes it superior to every other material for packing. What this is I do not know, but under its use the dressing is drier, there is less pus, and the whole case goes on in a more orderly and rapid manner. I like, also, to rub iodoform into the walls of these abscesses and into the sides of a fistula. Peroxide of hydrogen is one of the best antiseptics in these cases.

In regard to the bowels, I have a predilection for keeping them loose in all operations about the rectum, and very early give such drugs as will produce partially liquid stools. This gets rid of the great pain and the terrible anxiety of the patient looking forward to the movement of bowels that have been constipated for several days after an operation.

Repacking is important, but, in many cases, simple inspection, particularly in fistula, is all that is necessary to see that healing is progressing properly, and, if necessary, the wound can be painted with some stimulating solution, as a fifteen-grain solution of silver nitrate, balsam of Peru, etc. By careful watching, the painful process of repacking can often be avoided, although in some cases it will be required to secure uniform healing from the bottom.

Coming to fistula-in-ano, we should bear in mind that there are two great varieties ; one is the tubercular, and the other the simple or traumatic, where a small foreign body perforates the wall of the rectum and sets up inflammation in the cellular tissue. Probably, the ones that form slowly are simple, but may become infected with the tubercle bacillus. Granting that the large majority are tubercular, the treatment must be thorough and radical, in order to get rid of the tubercle bacillus.

I agree with Dr. Wharton in regard to the use of the ligature, that only cases involving high section of the bowel justify that method. By this method we are apt to overlook the branches, which are so common.

It is not always best to carry the director through the fistula and lay it open at once. It is sometimes better to dissect from the outer opening, treating the branches as they are met with, rather than to make the incision and have the field of operation obscured by blood.

The excision of the fistula has not proven a success. I have conversed with one or two men who had much to do with the introduction of this method, and they acknowledge that, after using it for several years, it has proved far less useful than had been hoped. There were but few cases in which excision could be done, and, of these few, the majority suppurated, and the edges of the wound had to be separated.

In fistulas that are tubercular, and, perhaps, in all, after thorough dissection and opening up all branches, I have wiped the wound out with pure caustic potash. That stops the hemorrhage, it kills the lining membrane throughout, and, perhaps, some healthy tissue, but I think that we get a more radical operation, particularly in tubercular cases. We must, however, be careful not to go too deep. After using the caustic potash, it is well to carry over the wound a sponge saturated with vinegar, in order to stop the action of the alkali. The indurated tissue can also be destroyed with the Paquelin cautery, but when this is used, the surrounding tissues must be very carefully protected from the radiating heat.

There are scarcely any fistulas that cannot be benefited by operation. There are many that cannot be cured, but all that I have seen have been benefited. I can recall several cases that are going about with a small sinus running high up, but yet are remarkably comfortable when contrasted with their former deplorable condition.

Dr. A. J. Downes: Neither of the gentlemen has referred to multiple fistulas. I recently had such a case in a tubercular woman forty-two years old. During the past ten years she had had four ischio-rectal abscesses that had opened, and numerous other phlegmons that had not opened. I operated on her four weeks ago at St. Joseph's Hospital. I found four fistulas. On the left, two,—one close to the external sphincter and entering the bowel behind the internal sphincter; the second, an inch and a quarter from the margin of the bowel and entering the bowel an inch and a half from the anal margin. On the right side there were two, whose external orifices were two inches apart, and an inch and a half from the margin of the bowel. In operating I cut through the left side at right angles to the sphincter, and on the other side made a wedge-shaped incision, extending above the openings in bowel, including both fistulas. I expected to cut the sphincter on this side at a subsequent operation. Sixteen days later I etherized and found that I had succeeded in closing the internal openings on the right side. I had thus succeeded in one operation in curing the four fistulas with but one incision through the sphincter, nor can I see why it should be necessary to even cut the sphincter twice in any case.

On two occasions in the office I have used the rubber ligature for fistula in otherwise healthy people, with excellent results.

Dr. J. M. Baldy: I have been somewhat surprised at the small amount of space that Dr. Wharton has given to excision of fistulas and to the utter condemnation of that treatment by Dr. Morton. My experience has been so contrary to this that it may be of interest to mention it. In past times I have treated fistulas by cutting and packing, and I have seen a number of cases in which non-control of the sphincter continued for a long time. I then gave excision a trial, and I have never in a single instance regretted its use, nor have I had a single failure in the ten to fifteen cases in which I have employed it in the last two years. In simple fistulas I have slit open the fistula, and with curette, scissors, or thin-bladed knife, removed the indurated tissue, and then have placed sutures buried completely around the fistula; and have finally closed the mucous membrane, from the bowel, with catgut sutures. Where there have been multiple sinuses, I have cut out all partitions between the sinuses. In one case the opening was one-half to three-quarters of an inch in width. I rubbed in a strong antiseptic solution, obtained primary union, and a perfect result. I remember a case in Trenton on whom a perineal operation had been done by a friend. This was followed by an ischio-rectal abscess with an opening into the rectum. The lady was also suffering from hemorrhoids. I laid the fistula open, exposing a cavity that looked most formidable. The slit in the bowel was probably two inches long. I curetted the cavity, rubbed in a strong bichloride solution, taking one-half to three-quarters of an hour, closed the wound with sutures, which did not go within half an inch of the bottom, whipped the bowel together with catgut, and closed it without drainage, at the same time removing four hemorrhoids. Ten days later, I removed the sutures, and in another ten days the patient was up and about, and the opening closed.

As I have said, in none of the cases operated on have I had cause to regret excision, and I should prefer it to all other methods where it was possible to introduce sutures, and nearly, if not fully, bury them all the way round.

In all my bowel operations I keep the bowels open from the first, and after the first passage the patients do not have much trouble. They are more comfortable than if they are permitted to have semi-hard discharges passing over tender surfaces.

Dr. WHARTON : I am glad that Dr. Morton has called attention to the importance of using the bichloride solution so that none of it is retained in the rectum. I cannot say why it is that iodoform gauze is so exceptionally good a dressing in these cases, but it is certain that it does better than any other material. I also use powdered iodoform.

Practically, the second dressing is not a repacking. I simply pass a piece of gauze to the bottom of the wound and allow it to project between the edges. I have also used the method referred to by Dr. Morton without repacking.

I use the caustic potash in many cases, particularly where the walls of the sinus are very dense, but neglected to mention it in the paper.

In the chronic cases that have been operated on many times, I think that the great secret of treatment is thorough curetting and free drainage. If the sinus can close so that pus can accumulate, the patient suffers. If the drainage is free, the patient is more comfortable.

In regard to double fistula, I spoke of this under the head of horse-shoe fistula, and recommended one division of the sphincter at a time.

Dr. Baldy's results have been very satisfactory. I have, myself, not had a very extensive experience with excision. I meet with many cases in which the operation could not be satisfactorily accomplished, and in these I prefer incision.

If the bowels are moved promptly after the operation, it is apt to disarrange the packing, and sometimes the first packing is important in controlling hemorrhage from the wound.

---

WHEN a boy really likes to go to church he should be taken to the doctor ; there is something wrong with him.—*St. Louis Medical Review.*

